# A Study on the Applicability of the Impact-Echo Test Using Semi-Supervised Learning Based on Dynamic Preconditions

**DOI:** 10.3390/s22155484

**Published:** 2022-07-22

**Authors:** Young-Geun Yoon, Chung-Min Kim, Tae-Keun Oh

**Affiliations:** Department of Safety Engineering, Incheon National University, Incheon 22012, Korea; yyg900@inu.ac.kr

**Keywords:** concrete, shallow delamination, air-coupled impact-echo, flexural mode, semi-supervised learning

## Abstract

The Impact-Echo (IE) test is an effective method for determining the presence, depth, and area of cracks in concrete as well as the dimensions of the sound concrete without defects. In addition, shallow delamination can be measured by confirming a flexural mode in the low-frequency region. Owing to the advancement of non-contact sensors and automated measurement equipment, the IE test can be measured at multiple points in a short period. To analyze and distinguish a large volume of data, applying supervised learning (SL) associated with various contemporary algorithms is necessary. However, SL has limitations due to the difficulty in accurate labeling for increased volumes of test data, and reflection of new specimen characteristics, and it is necessary to apply semi-supervised learning (SSL) to overcome them. This study analyzes the accuracy and evaluates the applicability of a model trained with SSL rather than SL using the data from the air-coupled IE test based on dynamic preconditions. For the detection of delamination defects, the dynamic behavior-based flexural mode was identified, and 21 features were extracted in the time and frequency domains. Three principal components (PCs) such as the real moment, real RMS, and imaginary moment were derived through principal component analysis (PCA). PCs were identical in slab, pavement, and deck. In the case of SSL considering a dynamic behavior, the accuracy increased by 7–8% compared with SL, and it could categorize good, fair, and poor status to a higher level for actual structures. The applicability of SSL to the IE test was confirmed, and because the crack progress varies under field conditions, other parameters must be considered in the future to reflect this.

## 1. Introduction

Over time, concrete buildings and civil infrastructure are subjected to continuous stochastic damage processes such as continuous loads, vibrations, and various degradation factors over a long period [1]. Their degree of damage has been attempted to be implemented as a general indicator, such as state grade by safety diagnosis within the perceived range of deterioration; however, several factors have been overlooked [2]. The most common cause of cracking in concrete is due to hydration, but this could be addressed with the development of new concrete and advancements in cooling systems [3,4]. However, various cracks beneath the surface in the concrete bridge deck, building slab, and pavements are vertical, or plate cracks caused primarily by corrosion of rebar inserted in concrete. Here, the presence of cracks in concrete does not always imply structural failure of reinforced concrete members [5]. However, an increase in humidity, temperature change, and harmful substances in concrete can accelerate various durability-related factors, and eventually, deterioration mechanisms such as corrosion, freeze–thaw, and carbonation can be expressed. Furthermore, cracks caused by continual corrosion ultimately led to delamination and exfoliation, compromising the structure’s integrity [6]. The delamination cracks in essential members of buildings and civil infrastructure, in particular, have a severe effect on the structural integrity and durability of structural members, threatening public safety [7]. In many countries, institutions in charge of infrastructure management spend a lot of money on maintaining existing structures rather than building new ones, and evaluating cracks in major concrete members is important for making proper maintenance decisions for concrete structures [8]. That is, maintaining the concrete members in sound conditions can extend its service life.

From this viewpoint, nondestructive testing and its fusion in the safety diagnosis of concrete structures have gained much popularity over the past 20 years [9,10]. However, there have been limited studies on the direct judgment of the nondestructive evaluation (NDE) based on various criteria of numerous data. Most NDE studies have focused on determining and verifying the extent of concrete deterioration using single or multiple NDE methods [11,12,13]. Furthermore, the NDE methods have relied on various physical phenomena, such as ultrasonic, electrical, strain, and thermal, to identify specific indicators of the degradation process.

The Impact-Echo (IE) test, developed by Carino et al. [14], is one of the most representative ways of determining damage to concrete structures. It is known as an effective ultrasonic NDE technology for detecting faults or defects inside and outside materials. The IE method can confirm the presence of voids, honeycombing, and delamination in platelike structures, such as concrete slabs and pavements [15]. Furthermore, the IE test effectively evaluates plate-type delamination cracks in concrete [16,17]. However, previous investigations in laboratory and field tests have revealed that conventional IE tests using a single sensor have many limitations. First, a single-channel measurement cannot provide concrete velocity information. The P-wave velocity is measured in many IE applications by testing in a structure region with known dimensions [18]. In addition, the speed may differ between the area where the speed is measured and where the actual test is conducted. Second, the IE test has limitations in the consistent measurement of the thickness mode. This arises from the disturbance of the noisy signal, which is not directly related to the mechanical properties of concrete [19]. Test results from single-channel measurements are insufficient to distinguish between delamination cracks and modes that are sufficiently important for extracting critical features of sound concrete. Third, the speed and data analysis of the IE test is slow when using the point test method.

To overcome these drawbacks, methods such as machine and deep learning have emerged, and through these methods, various features can be derived in addition to differences in dynamic components based on the dynamic or wave theory. Most of these methods are approaches based on supervised learning (SL). In addition, its application has a high potential to complement various weaknesses, such as nonlinearity and damage degree of concrete, and to check the current state [20]. Although actively using SL to detect defects in actual structures is required, manufacturing and testing concrete specimens depicting various types and types of defects are difficult. That is, realistically, there is a limit to producing a specimen that simulates all types of defects. Therefore, a method for supplementing the various types of data and labels is necessary, which is a problem with the existing SL method, and semi-supervised learning (SSL) can be an alternative [21]. Currently, the main field application of the SSL method is to build a judgment system for a large volume of data in the image field. It is also used in acoustic and ultrasonic data in the machine and language fields [22]. When SSL is employed, characteristics can be reflected even for new specimens, and it is possible to judge data that is difficult to label. Furthermore, the effectiveness is high as there is no need to label all data [23]. This study develops an SSL model that can quickly identify major signal features and consider the characteristics of new specimens using preconditions from the dynamics viewpoint for the signals obtained using the IE method, which is effective in identifying delamination cracks in flat concrete structures. In other words, SSL is applied in this study to solve the problem of insufficient IE data, which are difficult to manufacture; it is also difficult to consider various types of defects even when precisely manufactured. In addition, considering depth, area, and bending mode section, the classification accuracy of sound, fair, and delamination is improved when setting the preconditions for delamination defects. Furthermore, the possibility of using SSL for structural health monitoring of concrete is presented by comparing the SL and SSL analysis results.

Since the introduction of the IE method in the 1980s, it has been widely known as a representative nondestructive method for finding the integrity and defects of concrete members and structures [16]. Examples include the investigation of bridge deck plates [24], internal defects of partial shear connection slab [25], corrosion damage to reinforcing bars [26], and the condition of shotcrete defects [27]. Over the decades, improvements in the performance and capabilities of IE methods have been actively achieved. Kim et al. proposed a method for evaluating the P-wave velocity in determining concrete thickness using spectral analysis of surface waves (SASW) [18]. They assumed Poisson’s ratio, and the predicted surface wave velocity from SASW was converted to the P-wave velocity of the subject. Ryden and Park developed a fusion of multichannel surface waves (MASW) using the IE test method [28]. They used the MASW to analyze both transmitted and non-transmitted Lamb waves. In addition, they employed both amplitude and phase information from multichannel recordings to improve the identification of the thickness mode and selectively used it to predict the P-wave velocity. The main purpose of their study was to develop an improved IE test method [29].

Among these advancements have been advanced analysis goals, such as improved filtering of IE signals, development of signal processing techniques, and machine and deep learning. More specifically, it was primarily signal processing in the past, but recently, machine and deep learning methods have been employed. In signal processing, to examine the non-static characteristics of IE signals, time-frequency analysis such as short-time Fourier transform (STFT) [30], empirical mode decomposition (EMD), and Hilbert–Huang transform (HHT) have been used as an alternative to traditional fast Fourier transform (FFT) analysis [31,32,33]. Additionally, wavelet transform can show temporal and scale information simultaneously, making it easy to analyze periodic, instantaneous, and noisy times [34]. To increase the reliability of inference in using NDE techniques, researchers have adopted a data integration mechanism that integrates various NDEs for condition monitoring [35,36]. The evaluation of the condition of concrete structures using ground-penetrating radar (GPR) and infrared thermography (IRT) can provide valuable consideration [37,38]. The application of clustering and fuzzification, which fuses inferences from GPR and IRT, worked reasonably well to identify damaged areas. Since then, it has developed in the direction of modeling the condition grade of the structure using various data such as subjective (visual inspection) and objective (NDE surveys) results [39].

Recent studies [40,41] provide a deep overview of machine learning applications. Machine learning has been less explored compared with signal processing as a means of improving IE methods. Several studies have used machine learning, such as artificial neural networks [42] and support vector machines [43], to determine the internal defect of the IE signal through the Bayesian classifier [23]. The features used for classification were the spectra of IE obtained from accelerometers located on different specimen surfaces. They used principal component analysis (PCA) to reduce the feature dimension. Extreme learning machine (ELM), a special form of a feed-forward neural network, is one of the recent machine learning methods. Since its introduction in 2006, ELM has been used in computer vision [44], image processing [45], medication [46], and text understanding [47] as well as in IE [20].

However, proper labeling for the entire temporal domain is very expensive and time-consuming [48], and there are many cases with unknown defects in the mechanical system [49]. In other words, in practice, label information about the state of a system often does not exactly match the corresponding sensor data. For example, fully labeled datasets are obtained from laboratory or test runs, whereas unlabeled datasets are obtained from the same system in the field environment. Therefore, several unsupervised learning approaches have been introduced to leverage the advantages of both supervised and unsupervised learning [50,51]. Zhao et al. proposed an unsupervised learning classifier for detecting defects in solar photovoltaic arrays [52]. Using both supervised and unsupervised classifiers as an unsupervised learning method, they normalized and filtered measurements to classify defect data from other data. This method has exhibited good performance without labeling costs on continuous model updates. To extract meaningful vibration signals, an unsupervised classifier was applied along with kernel marginal Fisher analysis [53]. Relevant researchers have derived optimal low-dimensional features to optimize redundant information to discriminate meaningful signal behavior related to system state changes and to improve the discrimination performance of various bearing defect types. In the IE data analysis and interpretation, an unsupervised Bayesian classifier was developed to check the degree of damage in materials using IE signals, and it was extended to hierarchical clustering analysis. The model was trained using prefabricated defects with 10% learned labeling [54]. However, Igual’s study, based on the PCA of various wave paths for plate shape and cylinder in a cubic specimen, is closer to the resonance test of a cubic object than IE. On the other hand, in this study, the induction of flexural and thickness mode excitation in plate structures, derivation of features in the frequency domain, and in-depth analysis of the differences between SL and SSL for features of artificial delamination and real defects in real bridges were presented.

## 2. Theoretical Background

### 2.1. Impact-Echo

**Figure 1:**
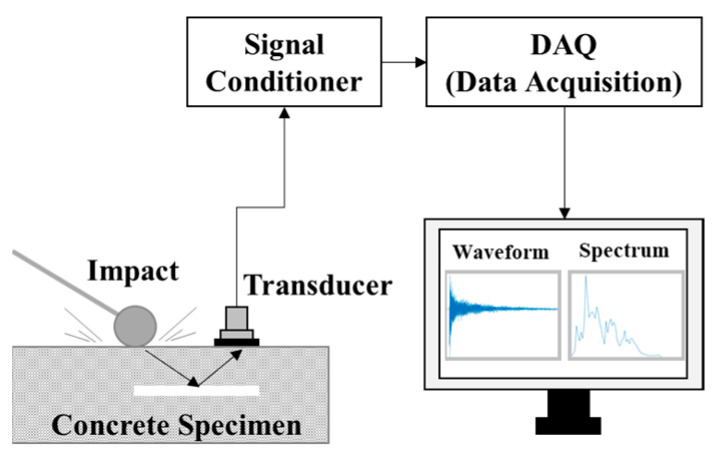
Schematic of Impact-Echo method.

The IE method was established as a reliable method to check dimensions or lamellar cracks in concrete slabs and pavements [55,56]. The basic principle of the IE method is based on the instantaneous resonance of a platelike structure subjected to mechanical impact. The instantaneous temporal response of a sound structure is measured by the response of displacement, velocity, and acceleration at a surface close to the resonance source. The obtained response signal is analyzed in the frequency domain using FFT, as shown in Figure 1. The amplitude and frequency values in the frequency domain for a specific resonance mode are used to calculate the presence or absence of delamination and the thickness of the slab [57]. For multiple reflected waves of the stress wave, the slab thickness *h*, the P-wave velocity *Cp*, and the peak frequency in the frequency spectrum are related to each other in the following Equation (1) [57]:

(1) $$h=\frac{\beta C_{P}}{2f},$$

where *β* is a shape factor of approximately 0.96 in an infinite plate structure.

The non-contact IE test system replaces the contact sensor with a non-contact sensor. The basic principle of the non-contact IE test method is to measure a leaky wave generated by a surface wave with a microphone. Various studies have shown that the non-contact IE data provide the same results as a conventional contact sensor [58]. The IE method is applied to locate defects with air below the test site and is not effective in the case of closed cracks [59]. For lamellar cracks parallel to the surface and having a large area, the depth can be estimated using Equation (1). For example, using Equation (1), when β is 0.96 and Cp = 4000 m/s in a plat and general concrete, the frequency of 5–10 mm delamination (lamellar crack) is approximately 19~39 kHz. Therefore, lamellar cracks can be found with a peak corresponding to the thickness mode in the range of 20 kHz or higher. However, it is difficult to implement the thickness mode if the crack size is small or not flat. Additionally, when the delamination crack is near the surface, (e.g., when the *a/h* value is large), it is difficult to distinguish the thickness mode because the bending mode is dominant [59].

### 2.2. Dynamic Behavior of Concrete Defects

**Figure 2:**
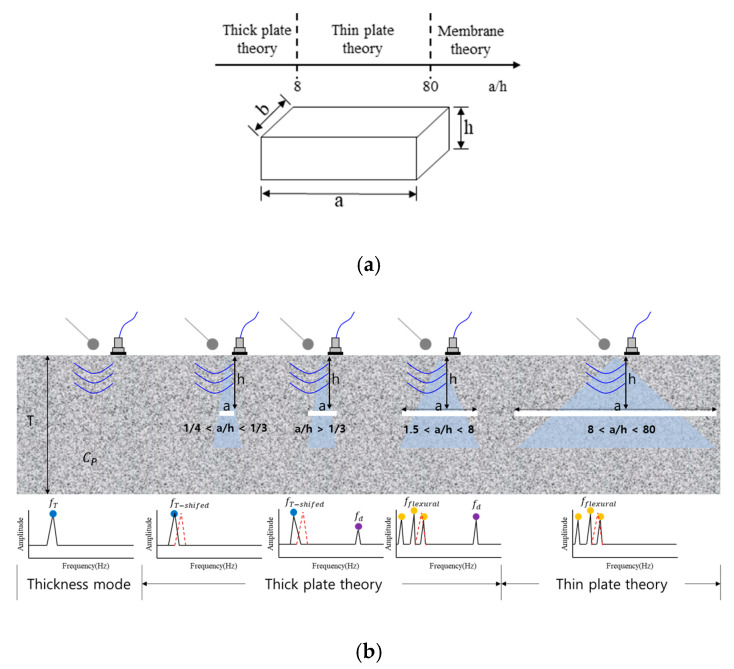
Criteria governing thick plate, thin plate, and membrane cases based on the side-to-thickness ratio *a*/*h* (*a* > *b*). (**a**) The plate theory range according to *a*/*h*; (**b**) the tendency of thickness and flexural mode according to *a*/*h*.

In the case of delamination cracks inside the structure in the IE method, plate theory can be applied by assuming the plate with the width (*a*) and depth (*h*) under a semi-clamped boundary condition over the horizontal crack. According to the plate theory, plates can be classified as thick plates, a thin plate, and a membrane, which can be classified as shown in Figure 2 using the plate width/thickness ratio (*a*/*h*) [60]. Generally, as the value of *a*/*h* decreases, (e.g., *a*/*h* < 10), the effect of shear deformation and rotational stiffness cannot be ignored, so the bending behavior of the plate is difficult to express numerically [61]. Since delamination cracks exist at various widths and depths, they can have various *a*/*h* values, and the relative dominance of the bending mode and Impact-Echo mode (thickness mode) changes according to the change in *a*/*h*. Generally, when the *a/h* value is large, the bending mode dominates, and the amplitude value of the thickness mode is quite small, making it difficult to measure. However, as the *a*/*h* value decreases, the size of the thickness mode starts to increase, and as the value approaches the thickness of the plate, the thickness mode becomes clear and can be easily distinguished from the values of other modes. It is important to study the dynamic behavior of the plate in the bending and the thickness modes, which are sensitive to changes in *a*/*h* values.

Although the frequency range by the classical IE method is extended up to 15 kHz (slab thickness 150–300 mm), the dominant frequency for delamination cracks is up to 6 kHz in the typical case of area dimensions (100–2000 mm) and depth (10–100 mm) ranges assuming a thin plate with the clamped boundary condition according to Equation (2) [60]. Additionally, using the IE test, the detectable area size of delamination should be greater than 160 × 160 mm at less than 6 kHz [62].

(2) $$\nabla^{2}\nabla^{2}w+\frac{\rho \partial^{2}w}{D\partial t^{2}}=0,$$

where *w = w(x, y, t)* is the transverse deflection, which corresponds to the thickness (‘*h*’ direction, as shown in Figure 2a; $\nabla^{2}$ is the two-dimensional differential Laplace operator ($\nabla^{2}=\partial^{2}/\partial x^{2}+\partial^{2}/\partial y^{2}$); $D=Eh^{3}/12\left(1-v^{2}\right)$ is the flexural rigidity; *E* is Young’s modulus; *h* is the plate thickness; *v* is the Poisson’s ratio; ρ is the mass density per unit area of plate surface; *t* is the time.

### 2.3. Principal Component Analysis

**Figure 3:**
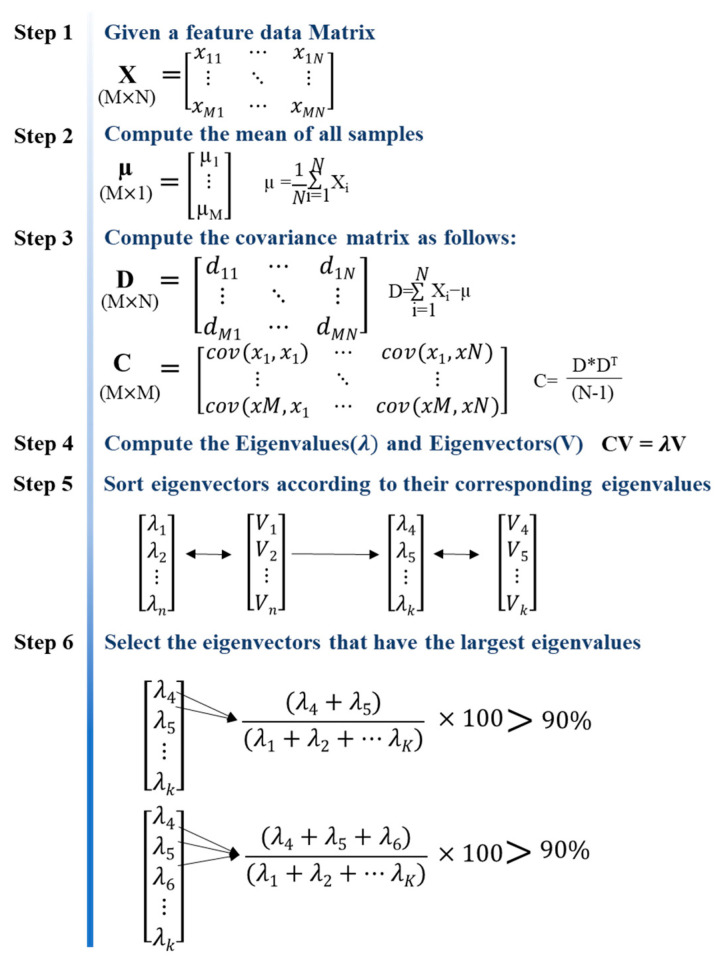
PCA procedure.

Principal component analysis is a method for reducing high-dimensional data with various variables to low-dimensional data. It was proposed by Pearson and later improved by Hotelling and Jolliffe to establish a modern theory [63,64]. PCA linearly transforms data into a new coordinate system such that when data are mapped to one axis, the axis with the largest variance is placed as the first principal component, and the second largest component is placed as the second principal component. Therefore, PCA is a method for extracting components that best represent the data distribution, and Figure 3 presents the procedure [65]. Specifically, in the first step, the feature data matrix X consists of a matrix having M × N data as a basic matrix. In the second step, the average (*μ*) of each row of the X matrix is calculated to construct the average matrix, and in the third step, the deviation matrix is constructed by subtracting the mean from the element values of the basic matrix X. Thereafter, in step 4, the covariance matrix may be derived by multiplying the deviation matrix by the transposed matrix (*D^T^*) of the deviation matrix. Additionally, eigenvector and a eigenvalue (*λ*) are calculated using the obtained covariance matrix, and the eigenvectors are arranged in ascending order according to the size of the corresponding eigenvalue to derive the principal component.

It is difficult to quantify the extent to which the theoretically occurring main frequency in the IE spectrum is shifted according to the degree of delamination defect. In this study, various features of the flexural mode were derived in a spectral region of a certain range by considering the dynamic characteristics in the IE spectrum, and PCA was employed to analyze them.

### 2.4. Semi-Supervised Learning

Semi-supervised learning (SSL) is a method that enables the effective use of unlabeled data in machine and deep learning. Chapelle et al. [66] summarized the assumptions that formed the basis for the successful application of SSL and provided a comprehensive introduction to various subjects. Van Engelen and Hoos [67] provided a review in the field of SSL for taxonomy. The approach to the behavioral recognition of forklifts is suitable within the framework of a wrapper method that uses unlabeled data associated with the virtual labeling step. In the wrapper method, a model is first formed through labeled data using SL, and unlabeled data are classified using this model.

**Figure 4:**
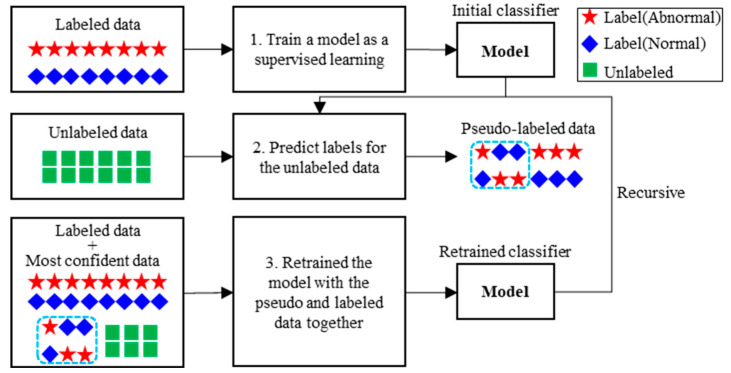
General framework of the self-training classifier.

In this study, instead of SL with high prediction probability, as presented in Figure 4, all virtual labeled data are used, and a pseudo-labeling process is used to learn new features rather than a trained follow-up model. This approach is comparable to the naive semi-supervised approach in that deep learning methods generalize better after training on noisy data [68].

The algorithm first trains on the labeled data through a designated classifier and then performs a label prediction on the unlabeled data. It checks the score for the prediction and, if it exceeds the predetermined ScoreThreshold, treats the prediction as the actual label for the next training cycle. This process is repeated until the label prediction converges. Each iteration of the algorithm performs label predictions on unlabeled observations and computes a score for these predictions. Unlabeled observations with a predicted score greater than or equal to the score threshold are treated as labeled observations in the next iteration.

## 3. Materials and Test Procedure

### 3.1. Materials and Preparation of Specimens

**Figure 5:**
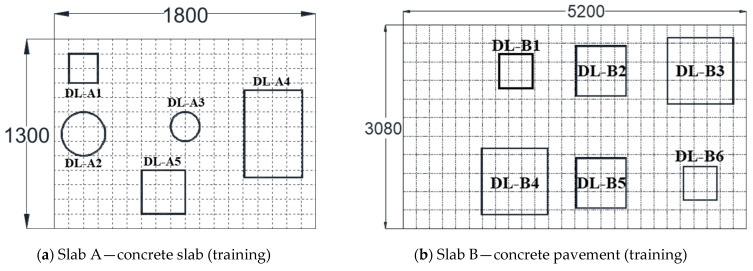

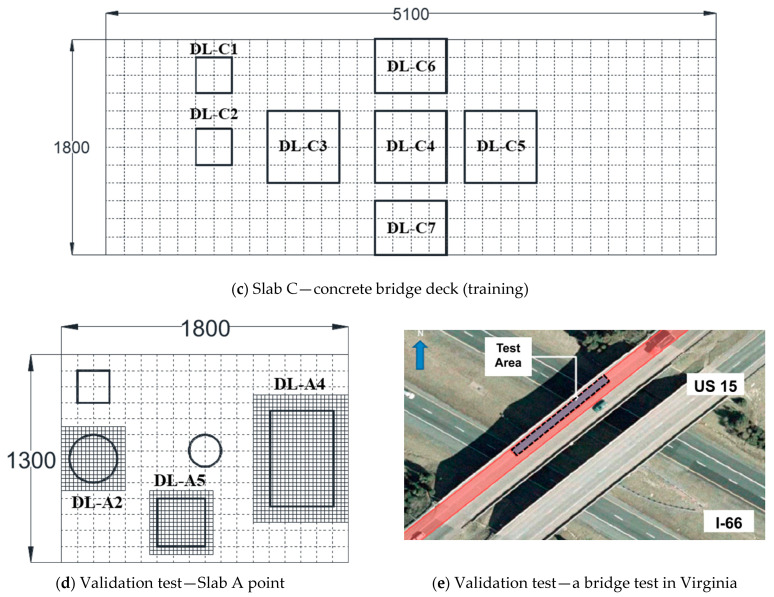
Test specimens for training and validation with SL and SSL.

To identify shallow delamination of concrete, air-coupled IE tests were conducted on sections of concrete slabs, pavements, and bridge decks with simulated artificial defects. The three specimens had various artificial defects and voids. Figure 5 presents a plan view of the three test specimens, and Table 1 shows the details. The concrete of all specimens had a 28-day compressive strength of 30–40 MPa, and the ultrasonic pulse velocity of mature concrete (measured according to ASTM C597 (ASTM 2009)) ranged from 4000 to 4200 m/s.

The slab was reinforced with two-dimensional and double rebars with depths of 60 and 200 mm. Slab A had artificial delamination and voids of various sizes and depths, as presented in Figure 5a. The double-layer plastic sheet and the flexible foam block simulate artificial rectangles (DL-A1, A4, and A5) and circular delamination (DL-A2 and A3), respectively. A grid of 10 × 10 cm^2^ test points was defined on the surface, and an air-coupled IE test of 266 points at the grid location was conducted. In Slab B, the unreinforced pavement section consisted of a thin layer of Portland cement concrete 50 mm deep on top of a thick (>150 mm) asphalt concrete base layer. The pavement contains six artificial delamination defects (double-layered polymer sheets) of various areas and depths, as presented in Figure 5b, located at the interface between the concrete and asphalt layers. The depth of the delamination defect is divided into 25 mm (DL-B1 and B2 and B3) and 50 mm (DL-B4, B5, and B6). The IE test grid is 20 × 28 cm^2^ in the longitudinal and transverse directions. Air-coupled IE data of 324 points were collected over a large area along a defined test grid. In Slab C of Figure 5c, the concrete deck’s length, width, and depth are 5.1 × 1.8 × 0.2 m^3^, respectively, to simulate a real-scale RC bridge. The bridge deck slab includes two uncoated steel reinforcement mats, 60 and 150 mm deep. Defects were simulated using embedded foam pieces of various sizes and depths. The shallow delamination (DL-C1 to C6) and deep delamination (DL-C7) had a depth of 60 and 150 mm, respectively. A 15 × 15 cm grid of test points was defined on the surface, and an air-coupled IE test of 455 points at the grid location was conducted. IE data of 1045 points were collected for the three slabs, which were used for training the SL and SSL models. For the primary performance verification of the additionally developed model, DL-A2, DL-A5, and DL-A6 of Slab A were remeasured with a detailed grid, as presented in Figure 5d. The Virginia bridge measurement data in Figure 5e was used for secondary verification to confirm the field applicability of the model collected from the artificial fault data.

### 3.2. IE Test Procedure

**Figure 6:**
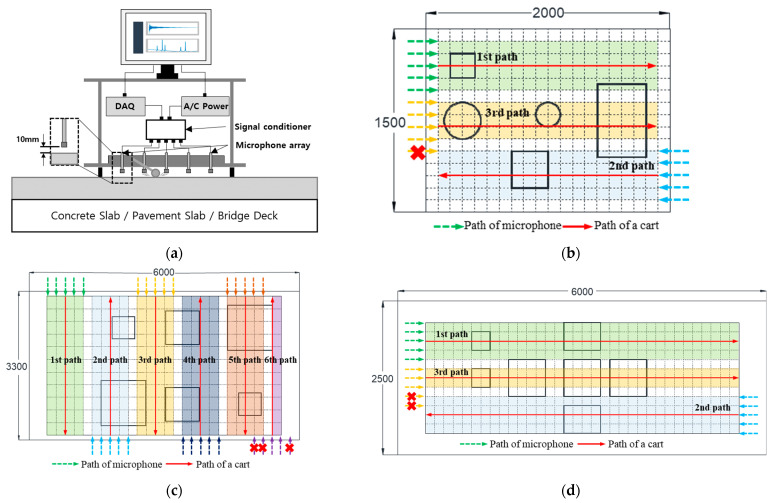
Test equipment and procedure: (**a**) test equipment; (**b**) test procedure for slab A; (**c**) test procedure for slab B; (**d**) test procedure for slab C.

A mobile microphone was installed near 10 mm of the concrete surface, as presented in Figure 6a, and elastic waves were generated and measured for effective dynamic response measurement. This study used a steel ball hammer with an 18 mm diameter to generate elastic waves on a concrete specimen. This hammer is suitable for generating signals of very low frequency (to 15 kHz). The sensor used for measuring the dynamic response of the concrete specimen was a dynamic microphone (SM58, Shure, IL, USA ) with a sensitivity of 1.85 mV/Pa at 1 kHz and an operating frequency range of 50 to 15 kHz. An IE test was conducted, as presented in Figure 6b, by placing five microphones of the same specification. Figure 6 presents the test hardware setup, and several microphone sensors are mounted on a mobile cart. In the case of Slabs A–C, the horizontal measurement distance was adjusted according to the width of each grid. In the case of Figure 5d, by hitting manually, the impact event is applied to individual sensors to obtain a single signal corresponding to the test point. The measured signal was amplified using a signal conditioner (PCB 482C16, PCB, NY, USA) and digitized by sampling at 1 MHz through an oscilloscope (NI-PXIe 6366). For normalization to the same energy, it was divided into the absolute value of the maximum value of the Rayleigh wave in the time domain and transformed into the frequency domain using the FFT algorithm [59].

## 4. Results and Discussion

### 4.1. Experimental Results of the Impact Resonance Test for Flexural and Thickness Modes

#### 4.1.1. Analysis of FFT Domains

Previously, studies on the occurrence of IE mode and flexural mode were conducted for plate-shaped (delamination) defects. If the flexural mode is not considered, detection of delamination defects based on the IE mode may be difficult due to the limitations of high-frequency generation and experimental errors. This study described various artificial delamination defects in the thick and thin plate theories for training SL and SSL models, and the flexural mode was intensively analyzed. In addition, according to the technological development trend, field applicability was increased using a non-contact microphone rather than an in-lab test using an accelerometer. First, *a/h* was calculated to confirm the occurrence of the flexural mode by type of detachment defect, and Table 2 presents a summary of the trend of dominant flexural mode. Here, based on *a/h*, “o” means dominant flexural mode, and “x” is non-dominant. When *a/h* is less than eight, such as DL-A1–A3, the thickness and flexural modes for defects may occur simultaneously among IE modes according to the thick plate theory. Additionally, when a/h is greater than eight, as in DL-A4 according to the thin plate theory, the flexural mode is expected to dominate. The thickness mode is expected to dominate, except for fabrication errors in the sound region and interfaces between the sound and delamination regions.

**Figure 7:**
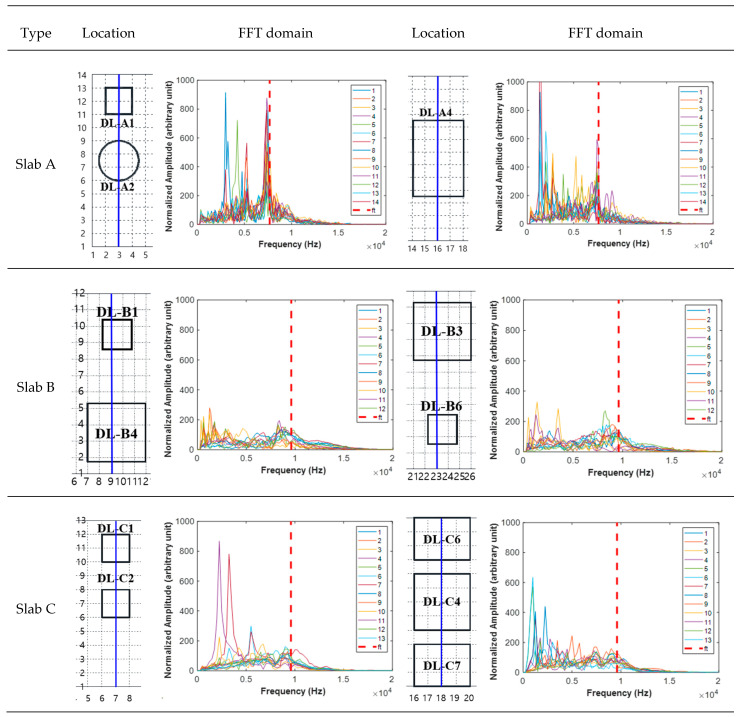
Representative spectrum by defect type and slab.

The *a/h* of delamination in this study ranges from 3 to 40 depending on the structural shape, and according to the theory of natural frequency, only a strong flexural mode occurs in the range of several hundred Hz to 5 kHz, or weak flexural and thickness modes coexist. Furthermore, the flexural mode does not appear in the sound region, and the thickness mode is dominant. It has been verified that strong frequencies within the range occur through IE test results. Figure 7 presents a plot of the FFT results for the sensor collected through the IE test, and the theoretical values for the possible thickness modes according to the thickness of the specimen are indicated. According to Equation (1), Slab A has a theoretical thickness mode of 7.68 kHz for thickness frequency (*f_t_*), whereas Slabs B and C are 9.6 kHz. Except for the area of delamination artificially depicted in the specimen, most showed similar results to the theoretical *ft*. However, there were cases in which other features appeared in some sound regions, which are analyzed to occur at the interface between delamination and sound due to insufficient fabrication. In the case of the thickness mode for delamination defects, it is analyzed that it occurs at 35 kHz or higher for the defect types in this study according to Equation (1). However, as there is a limit to having a frequency of 30 kHz or higher, this study used an 18 mm steel ball hammer (~15 kHz) to detect and analyze the defect through the generation of a flexural mode for the delamination defect.

In Figure 7, the IE test result of the sound region in the FFT domain of Slab A is analyzed as a peak comparable to the theoretical value for the thickness mode. The FFTs in DL-A2 and DL-A1 strongly generated flexural mode at 2 to 6 kHz. In addition, in the case of DL-A4, the flexural mode amplitude was strong and confirmed that it occurred more clearly. Slab B has a smaller amplitude than Slabs A and C, but it was confirmed that the flexural mode in the defect point and the thickness mode in the sound region could be distinguished when the maximum amplitude was 400. In Slab C, except for DL-C7, a flexural mode was clearly observed in the 1–5-kHz range. DL-C7 is an area where thick and flexural modes coexist with *a/h* of four; however, the flexural mode may not occur easily with 150 mm, the deepest defect tested. In the case of Slabs A and C, when looking at the amplitude of the flexural mode, the artificial defect seems to be described very well, and in the case of Slab B, the amplitude of the flexural mode is weak, so it is expected that the artificial defect production is somewhat insufficient. Although the characteristics of the flexural mode varied depending on the slab type (slab, pavement, or deck), the occurrence of the main flexural mode was confirmed below 5 kHz. Therefore, it is expected that using various features derivation and PCA in the time signal and FFT domains will allow for sufficient differentiation.

#### 4.1.2. Feature Extraction with PCA

**Figure 8:**
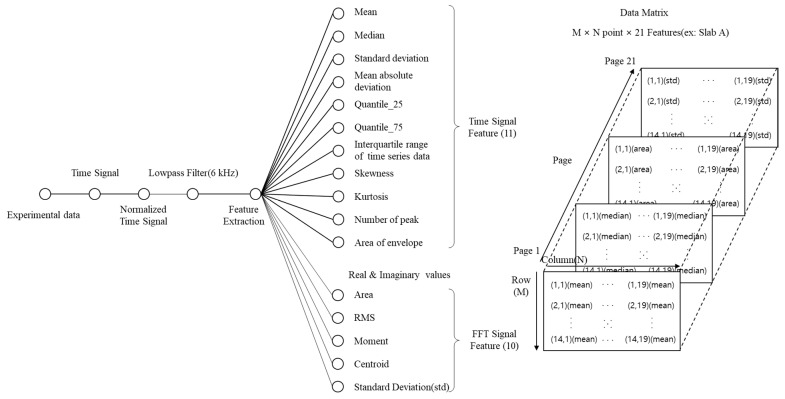
Signal processing and feature extraction procedure for PCA.

The existing IE spectrum analysis detects the shift or energy attenuation of the thickness mode or detects a new peak occurring at a high frequency according to the depth of the delamination defect according to Equation (1). However, if the physical properties of concrete change or some factors interfere with the propagation of elastic waves, it may be difficult to discriminate them using the theoretical formula. In the case of shallow defects, high-frequency generation due to general excitation is limited, and the shape of the flexural mode varies depending on the impact location; thus, an accurate analysis may be difficult. Rather than analyzing an inaccurate peak frequency of a spectrum, it is necessary to consider various features that can distinguish normality within a detectable frequency range. Moreover, analysis of uncertain results may require a professional ability to distinguish between defects and non-defects; however, through feature extraction and PCA, the distribution range of defects and sound regions can be distinguished, and quick and easy judgment is possible. In the dynamic response, according to the thickness of the three types of slabs considered in this study, the theoretically possible thickness mode is 6 kHz or higher, and the flexural mode, according to Equation (2), appears below 6 kHz. Therefore, a feature that maximizes the distinction between sound and defective is extracted by filtering the frequency in advance through a low-pass filter of 6 kHz. Twenty-one features are extracted through the procedure in Figure 8 for the time signal and FFT domain, and the extracted features were used as basic data for PCA.

**Figure 9:**
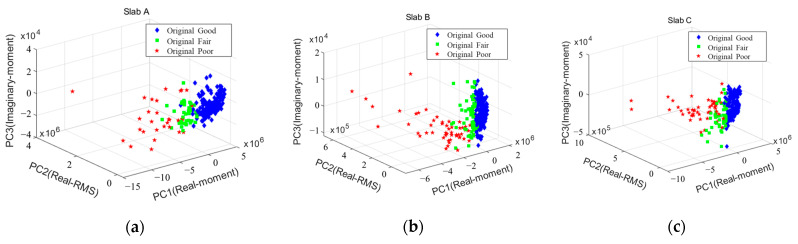
Three-dimensional plot of three principal components using PCA for each slab: (**a**) slab A, (**b**) slab B, (**c**) slab C.

Principal components (PCs) were derived through PCA for 21 features, PC1 (real moment), PC2 (root mean square, RMS), and PC3 (imaginary moment) were chosen as the main components with the largest variance in data, and comparable results were shown in the three slabs. Figure 9 presents the results of classification into three labels (good, fair, and poor) using PC1–3. The three types of labels are the intact part (good), the interface of the delamination (fair), and the upper part of the delamination (poor) in Slab A~C. Good data are distributed in the positive direction of PC1, and poor data are distributed in the negative direction of PC1. It is analyzed that good and poor data are generally clearly distinguished, and fair data are distributed as an intersection between them. This can be explained using the fact that the measurement position of the fair data is the interface region of the artificial defect and includes some data of the sound region due to an error in specimen manufacturing. In the case of fair or good data close to poor data, it is necessary to pay attention to the point where there is a possibility of a defect rather than to regard it as completely sound. By deriving the main features through PCA, all slabs, including Slab B, which was ambiguous in FFT, could be classified into three labels, and the data were used for training the SL and SSL models.

### 4.2. Prediction and Visualization of Controlled Delaminations with SL and SSL

#### 4.2.1. Analysis of SL Model Training and Prediction

**Figure 10:**
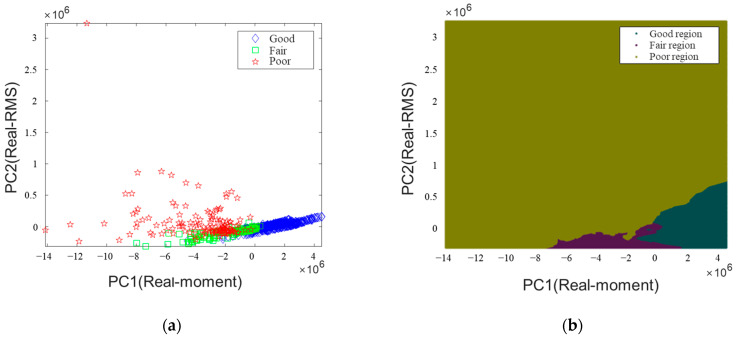

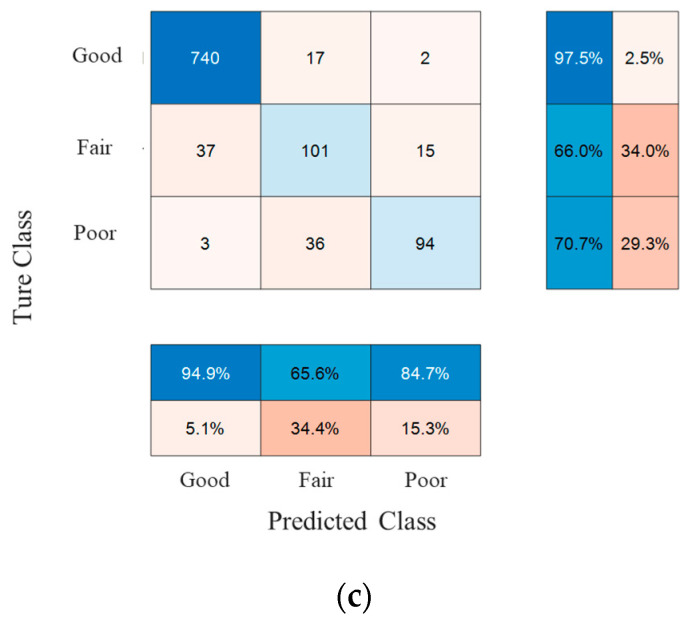
Predicted region and confusion chart through the SL model: (**a**) 2D plots of the full data used for training and validation; (**b**) regions of three labels predicted by SL; (**c**) prediction accuracy of the SL model.

Supervised learning was trained and verified using 1045 data from Slabs A, B, and C, as presented in Figure 10a. The ensemble method was used in Matlab 2022a, and K-nearest neighbors (KNN) were used as the detailed algorithm for training. Among the detailed parameters of the templateKNN, the number of neighboring data was set to 10, and the standardized was set to 1. This study has three classes, and the error-correcting output codes method using three binary classifiers was employed for multiclass training. Next, 10-fold cross-validation was performed using the “crossval” function to validate the trained SL model. Figure 10a presents a two-dimensional (2D) plot of the 1045 total data. The good data are concentrated in the lower-right corner, which means that it is of different types of slabs that are not very different and are similar. Poor data were not clustered and were distributed over a wide area. This is because it has different features according to the *a/h* of delamination. The actual model was trained using PC1–3 derived earlier, but predicting the three-dimensional area uses excessive memory; thus, Figure 10b presents a 2D plot. Figure 10c shows that the model’s accuracy trained and verified through SL was 89.47%. The general SL method has limitations in additional data correction as it predicts new data based on the previously trained data. Concrete often suffers from manufacturing errors, and there may be errors in the label data used at the beginning. Therefore, if there is a large volume of data with an accurate label, it may be effective to build a model using the SSL method.

#### 4.2.2. Analysis of SSL Model Training and Prediction

**Figure 11:**
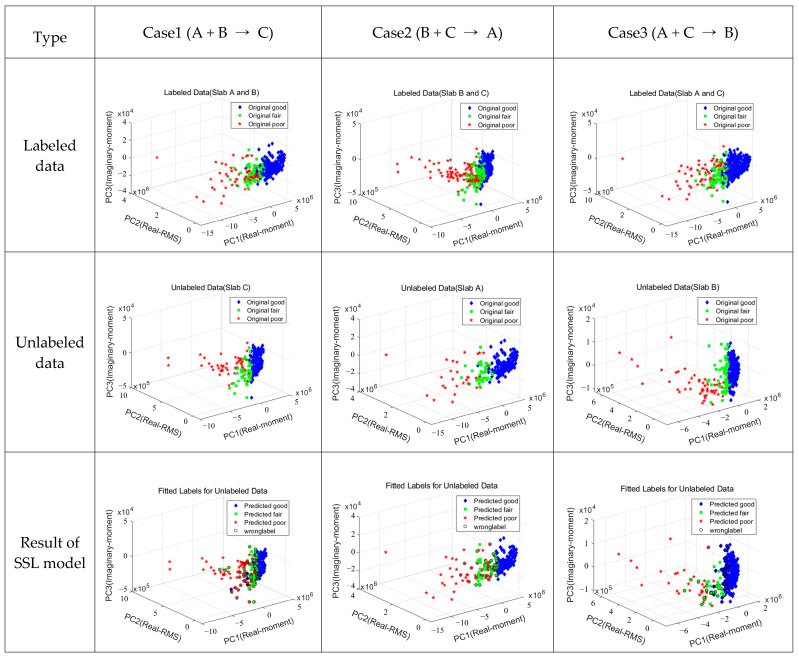
SSL model accuracy analysis by combination.

The SSL method is employed for mixing labeled and unlabeled data. First, an SL model is developed using the labeled data, and the label of unlabeled data is fitted using the created model. In this process, the optimal label is assigned through 1000 iterations. Therefore, it is important to use accurate label data. In this study, as presented in Figure 11, the SL model based on the KNN algorithm was trained for Cases 1 to 3, and the optimal model was selected by fitting unlabeled data with SSL. In Case 1, a training model was created using Slabs A and B, and an SSL model was developed using Slab C as unlabeled data. For unlabeled data, accuracy was analyzed as 78.24% (356/455). In Case 2, the SL training model was developed for Slabs B and C, and the SSL model was developed for Slab A. Among 266 data, the number of erroneous label data was 43, which had an accuracy of 83.83%. Case 2 analyzed that the unlabeled data were predicted with higher accuracy as the labeled data were similar, the intersections were small, and the distinction was somewhat clear. In Case 3, a training model was built with Slabs A and C, and Slab B was fitted. The accuracy was 77.16% (250/324), which is the lowest among the three cases. The distinction between fair and poor data is ambiguous in the distribution of labeled data, and it is analyzed that the dispersion of good data is rather large. When fitting with SSL using the data of Slab B, most of the fair data are analyzed as fitting as good or poor data.

As a result of Cases 1 to 3, it was analyzed that Case 2 could fit unlabeled data with high accuracy due to the high data density for each label. The SSL model shows lower accuracy than the SL model; however, as this accuracy may be due to an initial set incorrectly labeled, further verification comparing the performance of the developed SL and SSL models is required. Therefore, in this study, two validation sets were composed by testing for three defects (DL-A2, A4, and A5) in Slab A and the actual Virginia bridge in Figure 5d,e. The performances of the two validation sets were compared by predicting the SL and SSL models.

#### 4.2.3. C-Scan Images Predicted with SL and SSL for Principal Components

**Figure 12:**
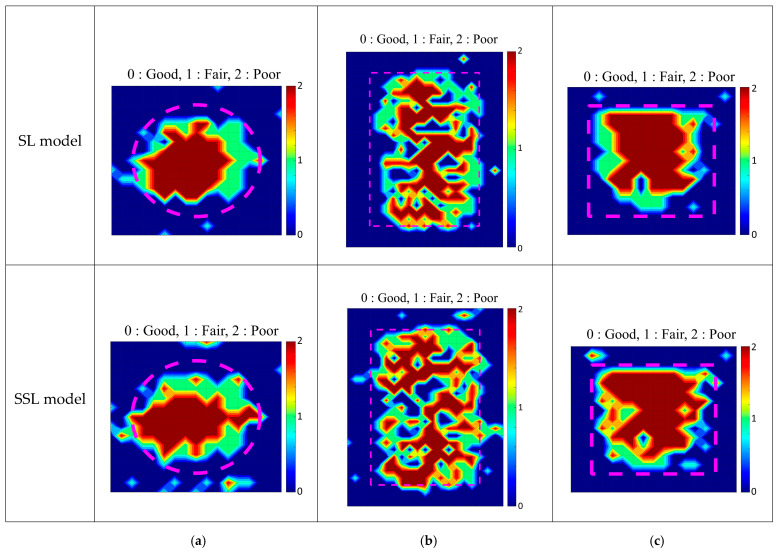
First performance verification of the SL and SSL models: (**a**) DL-A2, (**b**) DL-A4, (**c**) DL-A5.

Three defects in Figure 5d were remeasured with finer grids for the first performance verification of the developed SL and SSL models. For the remeasured data, features were extracted according to the procedure in Figure 8, and PC1, PC2, and PC3 were included for each point. Three extracted features were used as input data for the SL and SSL models, and the labels for each point in the grid were predicted. Figure 12 presents the C-scan image using the predicted results based on the two models. In Figure 12a, the accuracy of the circular defect of the SL model is about 70%, whereas the SSL model is about 78%. Furthermore, in Figure 12b,c, the SSL model showed higher accuracy than the SL model, and the performance of the SSL model on new data was analyzed to be similar to or slightly higher than that of the SL model. The SSL model is more accurate as it optimizes the error of ambiguous label data caused by the boundary of artificial defects and manufacturing errors through self-training according to smoothness and low-density assumptions. In this regard, the two models developed through Slabs A, B, and C can verify the equivalent performance for the three defects of Slab A. However, it is necessary to confirm the applicability of the actual structure due to data features of artificial defects for the training model and primary verification. Therefore, the model’s performance from artificial defects was further verified using the actual bridge test data in Virginia.

### 4.3. Field Application and Validation of Two Methods

**Figure 13:**
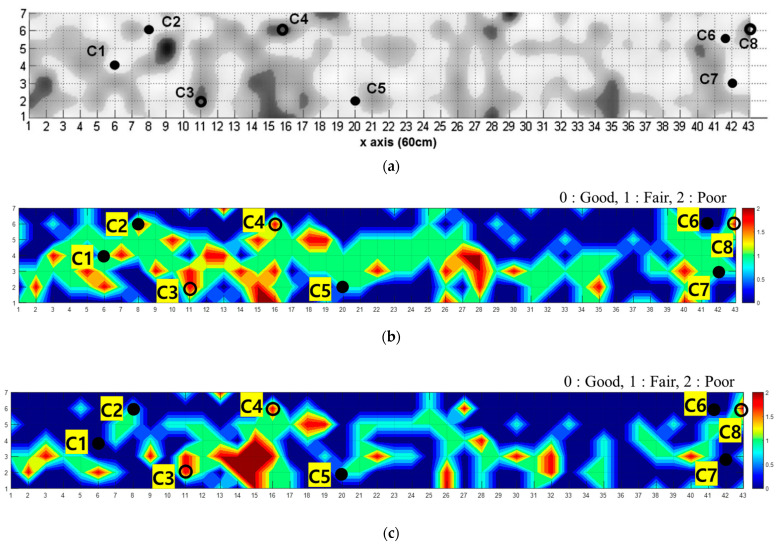
C-scan images for IE data collected from the bridge deck; spectral data are displayed up to 6 kHz. The closed and open circles are the positions of the good and poor cores, respectively. (**a**) Overlapped images of four NDT results analyzed by experts (image courtesy: Taekeun Oh), (**b**) prediction result of the SL model, (**c**) prediction result of the SSL model.

**Figure 14:**
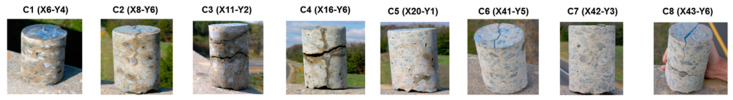
Eight drilled core samples, C3, C4, and C8, contain horizontal delamination at the top bar depth (image credit: Nenad Gucunski).

Additional analysis was conducted to confirm the applicability of the developed model to the actual bridge. Figure 5e presents the location where the actual bridge data were obtained. Oh and Popovics [62] conducted an advanced analysis of the measured results using four NDT methods: IE with Prototype A, IE with Prototype B, infrared, and sounding. Figure 13a presents an overlapped C-scan plot, and the results were verified through a core test, as shown in Figure 14. Eight core samples were drilled, consisting of five good and three delaminated. In this study, IE data for Prototype A [62] among the data collected by Oh and Popovics was used for the secondary performance verification of the SL and SSL models. According to the procedure in Figure 8, three main components from the data were derived, and these were used as input data. Figure 13b presents the result predicted using the SL model and shows that most of the region was predicted to contain defects. Here, the core samples C3(11, 2), C4(16, 6), and C8(43, 6) showed a delaminated region as in the previous analyses, but the expected sound region except for C6 was analyzed as fairs. It is analyzed that the prediction accuracy is less. Figure 13c presents the result predicted by the SSL model, similar to Figure 13a. C3, C4, and C8, which are delamination defect parts, were predicted as delaminated regions, and other sound regions, except for C2, were predicted with the same results. Specifically, in Table 3, The SSL model is more accurate than the SL model for the actual structure because when new specimen data is added, the physical and dynamic characteristics of the structure are reflected and optimized through the model update. Thus, the SSL model is sufficient for the concrete field through primary (for artificial defect) and secondary (for real bridge) verifications.

### 4.4. Flowchart for Using the SSL Model in a Concrete Field

**Figure 15:**
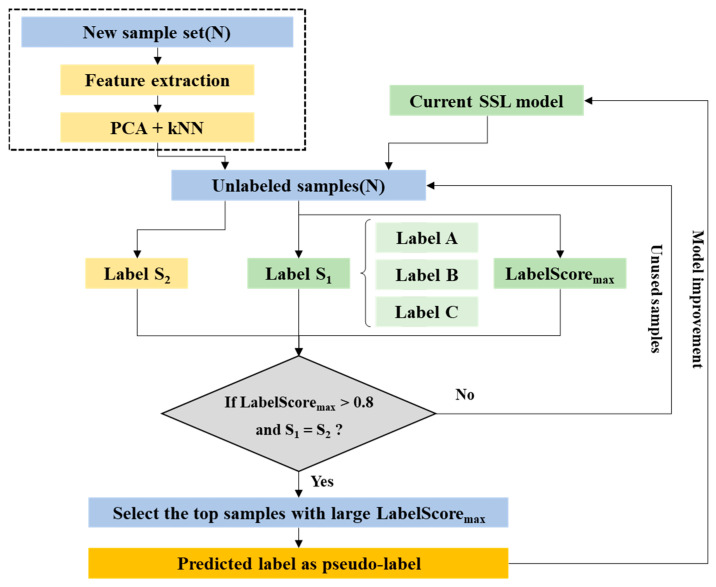
Proposed flowchart to optimize the IE data of concrete specimens.

The conventional SSL method has been applied to reduce the labeling effort in the field of image deep learning, which requires a large dataset. However, specifying labeling through a core test requires more effort and cost in the nondestructive test for concrete structures. Furthermore, a model developed with little labeling data may have less applicability to actual structures. Therefore, in this study, by applying the SSL method to minimize this problem, an SSL model was developed using labeled data with high accuracy, and the applicability by predicting the condition of the actual structure was verified. As the SSL model can be continuously improved using new unlabeled data, the proposed flowchart in Figure 15 can be effective for the condition evaluation of new structures. In the future, the SSL model should be improved with more categories of status other than good, fair, and poor through the continuous influx of new data.

Various research progressed on the utilization of the SL method using the existing IE test. However, these studies can be applied to concrete slabs under specific conditions, and their use is limited due to differences in the characteristics of artificial specimens and actual structures. In addition, it is difficult to reflect the new characteristics of the SL model developed in the artificial specimen after being developed. However, this study clearly presented the thickness mode or the flexural mode on delamination by the reflection of the body wave of classical IE, unlike previous similar studies. Afterward, it is meaningful that various types of concrete (slab, pavement, bridge deck) with different thicknesses and conditions were extracted simultaneously through PCA. The application of SSL to the extracted features has been verified through the results of Figure 13 and Table 3 to prove that it can be applied to real structures that are difficult to specify due to limitations in experiments and core tests. The SSL method has the advantage that the model is upgraded by continuously reflecting the characteristics of new specimens and actual structures. Therefore, it can contribute to the monitoring of structures through the application of SSL to concrete, which has limitations in specimen production and core testing.

## 5. Conclusions

Cost-effective and optimal maintenance and repair methods require information on material defects and deterioration conditions. Although the IE method effectively identifies internal defects of concrete, it requires expertise to analyze the signal. In this regard, this study analyzed the main features derived from the PCA for the pattern such as peak, energy area, and moment in the frequency domain of IE signals and proposed a semi-supervised detection model that can classify the presence or absence of defects in platelike structures, such as concrete slabs, pavements, and bridge decks, into three categories: good, fair, and poor. The SSL method has the advantage of updating the properties of new specimens and judging signals that are not appropriately labeled or that are difficult to label. As such, the novelty of this study can be summarized as follows:

- The limitations of previous studies that could not reflect various physical characteristics from valuable cases without synthetic data were resolved using verified reliable data.
- PCA efficiently and clearly extracted frequency domain features from typical IE data that reflect dynamic behavior based on repetitive body waves.
- Non-experts can readily use a conventional SSL algorithm, which can also be applied to actual bridge data.
- Moreover, an SSL model was developed in advance in this study using the specimens with simulated defects as unlabeled data, the unlabeled field data were verified, and the following summaries were derived.
- In comparison with the previous model, which used the entire frequency domain, an algorithm that can quickly and accurately determine the presence or absence of defects through dynamic preconditions was proposed.
- Compared with SL, the proposed SSL model can accurately determine the presence or absence of defects by about 7–8% or more through domain correction of inaccurate label data and updating of new specimen characteristics.
- In the future, the field data that is difficult to label can be applied by reflecting the characteristics of the new specimen.

Additionally, while the main purpose of this study has been achieved, further study is required to overcome various limitations. The detection performance of the current method requires additional measures to consider the field conditions, such as concrete strength and modulus, crack distribution, and defect severity. A more detailed study into whether other features in the time or frequency domain can perform early detection of the defect propagation and the development of various methodologies to improve the accuracy of unsupervised learning is required. If the limitations of these studies are minimized, the SSL method can be an effective method for nondestructive methods based on mechanical waves, such as IE, SASW, and MASW.

## Figures and Tables

**Table 1:** Attributes of specimens depicting artificial defects used for the training and validation of SL and SSL models.

Slab	Delamination	Width (mm)	Height (mm)	Depth (mm)	Type
Slab A	DL-A1	200	200	60	Plastic sheet
	DL-A2	ø300	-	60	Soft form
	DL-A3	ø200	-	60	Soft form
	DL-A4	400	600	60	Plastic sheet
	DL-A5	300	300	60	Plastic sheet
Slab B	DL-B1	500	500	25	Plastic sheet
	DL-B2	750	750	25	Plastic sheet
	DL-B3	1000	1000	25	Plastic sheet
	DL-B4	1000	1000	50	Plastic sheet
	DL-B5	750	750	50	Plastic sheet
	DL-B6	500	500	50	Plastic sheet
Slab C	DL-C1	300	300	65	Thin form (2 mm)
	DL-C2	300	300	65	Thin form (1 mm)
	DL-C3	600	600	65	Thin form (1 mm)
	DL-C4	600	600	65	Thin form (2 mm)
	DL-C5	600	600	65	Thin form (2 mm)
	DL-C6	600	450	65	Thin form (2 mm)
	DL-C7	600	450	150	Thin form (1 mm)

**Table 2:** Dominant flexural mode according to *a/h* by defect type.

Type	*a/h*	Dominant Flexural Mode	Type	*a/h*	Dominant Flexural Mode
Sound region	-	x	-	-	-
DL-A1	3.33	x	DL-A2	5	x
DL-A3	3.33	x	DL-A4	10	o
DL-A5	5	x	DL-B1	20	o
DL-B2	30	o	DL-B3	40	o
DL-B4	20	o	DL-B5	15	o
DL-B6	10	o	DL-C1	4.62	x
DL-C2	4.62	x	DL-C3	9.23	o
DL-C4	9.23	o	DL-C5	9.23	o
DL-C6	9.23	o	DL-C7	4	x

**Table 3:** Comparison of core test and prediction results.

Types	C1	C2	C3	C4	C5	C6	C7	C8
Oh and Popovics	Good	Good	Poor	Poor	Fair	Good	Good	Poor
SL model	Fair	Poor	Poor	Poor	Fair	Good	Fair	Poor
SSL model	Good	Fair	Poor	Poor	Fair	Good	Good	Poor

## Data Availability

The data presented in this study are available upon request from the corresponding author.

## Nomenclature

ELM	Extreme learning machine
EMD	Empirical mode decomposition
FFT	Fast Fourier transform
GPR	Ground penetrating radar
HHT	Hilbert–Huang Transform
IE	Impact Echo
IRT	Infrared thermography
MASW	Multichannel Analysis of Surface Waves
NDE	Nondestructive evaluation
PC	Principal component
PCA	Principal component analysis
RMS	Root mean square
SASW	Spectral Analysis of Surface Waves
SL	Supervised learning
SSL	Semi supervised learning
STFT	Short-time Fourier transform
*a*	Plate width
*b*	Plate height
*Cp*	P-wave velocity
*D^T^*	Transpose matrix
*E*	Young’s modulus
*f*	Frequency
*h*	Plate thickness
*t*	Time
*v*	Poisson’s ratio
$\nabla^{2}$	The two-dimensional differential Laplace operator
*β*	Shape factor
μ	Average
ρ	Mass density per unit area of plate surface
*λ*	Eigenvalue
